# Assessment of postpartum haemorrhage for placenta accreta: Is measurement of myometrium thickness and dark intraplacental bands using MRI helpful?

**DOI:** 10.1186/s12880-022-00906-2

**Published:** 2022-10-17

**Authors:** Xinyi Chen, Ying Ming, Han Xu, Yinghui Xin, Lin Yang, Zhiling Liu, Yuqing Han, Zhaoqin Huang, Qingwei Liu, Jie Zhang

**Affiliations:** 1 Department of Radiology, Shandong Provincial Hospital Affiliated to Shandong University, Jinan, Shandong China; 2 Department of Radiology, Shandong Provincial Hospital Affiliated to Shandong First Medical University, No. 324, Jingwu Road, Huaiyin District, Jinan, 250012 Shandong China; 3 Department of Radiology, Peking Union Medical College Hospital, Peking Union Medical College, Chinese Academy of Medical Sciences, Shuaifuyuan No. 1, Wangfujing Street, Dongcheng District, Beijing, 100730 China

**Keywords:** Magnetic resonance imaging, Placenta accreta spectrum, Volume of postpartum haemorrhage

## Abstract

**Background:**

This study aimed to investigate the predictive values of magnetic resonance imaging (MRI) myometrial thickness grading and dark intraplacental band (DIB) volumetry for blood loss in patients with placenta accreta spectrum (PAS).

**Methods:**

Images and clinical data were acquired from patients who underwent placenta MRI examinations and were diagnosed with PAS from March 2015 to January 2021. Two radiologists jointly diagnosed, processed, and analysed the MR images of each patient. The analysis included MRI-based determination of placental attachment, as well as myometrial thickness grading and DIB volumetry. The patients included in the study were divided into three groups according to the estimated blood loss volume: in the general blood loss (GBL) group, the estimated blood loss volume was < 1000 ml; in the massive blood loss (MBL) group, the estimated blood loss volume was ≥ 1000 ml and < 2000 ml; and in the extremely massive blood loss (ex-MBL) group, the estimated blood loss volume was ≥ 2000 ml. The categorical, normally distributed, and non-normally distributed data were respectively analysed by the Chi-square, single-factor analysis of variance, and Kruskal–Wallis tests, respectively. The verification of correlation was completed by Spearman correlation analysis. The evaluation capabilities of indicators were assessed using receiver operating characteristic curves.

**Results:**

Among 75 patients, 25 were included in the GBL group, 26 in the MBL group, and 24 in the ex-MBL group. A significant negative correlation was observed between the grade of myometrial thickness and the estimated blood loss (*P* < 0.001, ρ = − 0.604). There was a significant positive correlation between the volume of the DIB and the estimated blood loss (*P* < 0.001, ρ = 0.653). The areas under the receiver operating characteristic curve of the two MRI features for predicting blood loss ≥ 2000 ml were 0.776 and 0.897, respectively.

**Conclusions:**

The grading and volumetric MRI features, myometrial thickness, and volume of DIB, can be used as good prediction indicators of the risk of postpartum haemorrhage in patients with PAS.

## Background

Placenta accreta spectrum (PAS) is defined as the invasion of the chorion into the myometrium, and includes three different invasion degrees: placenta accreta, placenta increta, and placenta percreta [1, 2]. PAS leads to the failure of placental separation from the uterus after mechanical or surgical procedures without manual removal, and may be accompanied by different degrees of severity of haemorrhage. PAS is known as a risk factor for postpartum haemorrhage (PPH); however, the bleeding severity varies among patients with diagnosed PAS.

Prenatal ultrasound (US) is the modality of choice in diagnosing PAS because of its availability and ease of performance. Magnetic resonance imaging (MRI) enables multi-plane imaging of larger windows and has superior soft tissue resolution. Therefore, some studies suggest that MRI has greater sensitivity and specificity for PAS diagnosis compared to that of US, especially in delineating the degree of placental invasion [3, 4]. However, some studies reported that the PAS diagnostic performance of MRI was similar to that of US [5, 6]. MRI was performed as a secondary imaging tool in women who had already been screened for suspected PAS on US, and may thus not reflect its actual diagnostic performance in detecting PAS [7]. Especially in subjects who are planned to undergo MRI, MRI studies on PAS have recently focused more on the evaluation of relevant MR features to determine the degree of placental invasion in PAS patients [8], as well as in the prediction of the bleeding risk and the adverse outcomes of the maternal caesarean section (CS) [9–11].

Research of the previous literature, especially systematic reviews and meta-analyses, has revealed two MRI features which have attracted our attention: myometrial thinning (or focal interruption) and transverse relaxation (T2)-weighted imaging (T2WI) dark intraplacental bands (DIB). The superiority of the two features is that the former is a direct MRI sign indicative of PAS, while the latter is an indirect MRI sign of suspected PAS, with relatively higher sensitivity and specificity [6, 12–14]. The two features also appeared more frequently in PAS patients than that of other abnormal MRI signs, such as bladder tenting [15]. We speculated that the two MRI features might contribute to the prediction of severe PPH in PAS patients.

At the same time, we hypothesised that a numerical approach in terms of MRI myometrial thickness grading and DIB volumetry could be more accurate for PPH risk prediction rather than describing exclusively the presence of the two MRI features.

## Method

### Patients

This study was approved by the institutional board of Shandong Provincial Hospital. Individual consent for this retrospective analysis was waived.

From March 2015 to January 2021, 75 pregnant patients who underwent MRI examination and met the following standards were enrolled: *(1) patients who were confirmed as PAS by intraoperative or pathological diagnosis, and whose degree of placental invasion could be determined; (2) those who underwent placental MRI beyond the third trimester (gestational week 28) with complete axial, coronal, and sagittal images; and (3) those whose foetus was a singleton*. The exclusion criteria were: *(1) lack of complete personal clinical data; (2) the puerpera had other placental or uterine lesions; and (3) those who underwent labour induction rather than caesarean section (CS) due to stillbirth during pregnancy.*

In total, 125 pregnant women underwent MRI owing to suspected placental implantations. These patients had common suspicious clinical and ultrasound PAS factors. They received the advice for undergoing MRI examinations from an obstetrician.

Fifty of the 125 women were excluded, including 28 puerpera diagnosed with no placenta accreta during CS, 14 patients without complete electronic medical records, four patients who had only imaging data for postpartum placental implantation, two patients who had early caesarean scar pregnancy and two patients who underwent induction surgery due to stillbirth. Finally, 75 patients aged 26 to 43 (33.7 ± 4.3) years were included.

Clinical data of patients are shown in Table 1. The general clinical data included maternal age, gestational age, time from MRI scanning to CS, history of CS, and history of abortion. Data related to anaemia and coagulation function before CS included red blood cell (RBC) counts, haematocrit, haemoglobin, and activated partial thromboplastin time (APTT).

**Table 1:** Clinical information of patients

Information of patients		Group of GBL(25)	Group of MBL(26)	Group of ex-MBL(24)	*P* value
General information	Age (years)	33.0 ± 4.6	33.6 ± 3.5	34.4 ± 4.8	0.534
	Gestational age (day)	245.2 ± 16.7	249.1 ± 13.3	248.6 ± 15.0	0.619
	Time from MR scanning to CS (day)	10.24 ± 9.3	7.9 ± 8.9	11.1 ± 11.6	0.485
Anemia and coagulation function	RBC count (10^12/L)	3.8 ± 0.4	3.9 ± 0.5	3.7 ± 0.7	0.688
	Hematocrit (%)	34.1 ± 4.0	34.2 ± 4.9	33.0 ± 5.1	0.615
	Hemoglobin (g/L)	112.1 ± 14.0	110.6 ± 15.2	108.6 ± 16.5	0.725
	APTT (s)	30.3 (25.7,34.4)	31.6 (27.4,33.9)	32.2 (31.3,35.3)	0.140
History of CS	Zero	2	2	2	0.420
	Once	20	17	12	
	Twice	2	6	8	
	Three times	1	1	2	
History of abortions	Zero	7	10	5	0.780
	Once	10	7	9	
	Twice	6	7	9	
	≥ Three times	2	2	1	
Hemostasis measures	Prophylactic balloon occlusion	2	5	12	0.014
	Uterine arterial ligation	17	17	10	
	Uterine full/subtotal resection	0	2	3	
RBC transfusion (u)		0 (0,3.25)	4.00 (2.00,4.00)	6.50 (4.00,9.50)	< 0.001

*GBL* general blood loss, *MBL* massive blood loss, *ex-MBL* extremely massive blood loss, *MR* magnetic resonance, *CS* cesarean section, *RBC* red blood cell, *APTT* activated partial thromboplastin time, *u* unit

*Normally distributed data:* mean value ± standard deviation; *Non-normally distributed data:* median (first quartile, third quartile)

The diagnostic criteria for PAS were based on the diagnosis during operation and the pathological examination after CS. The intraoperative state of the placenta was observed by one obstetrician involved in the whole process and was reflected in the surgical records. Patients who underwent hysterectomy and completed postoperative pathological examination received a PAS diagnosis from the pathological assessment of the sections (five patients). Other patients were diagnosed based on both intraoperative placental adhesion (fifty-seven patients) and pathological examination of the delivered placenta (thirteen patients). The depth of PAS invasion included three different degrees: (1) placenta accreta, (2) placenta increta, and (3) placenta percreta.

The volume of estimated blood loss (EBL) of each patient was assessed by an experienced obstetric expert. EBL was estimated and documented by an expert after the surgical procedure. The data used to assess EBL included the volume of fluid in the negative pressure aspirator, and the weight of the gauze. The fluid in the aspirator was a mixture of blood and liquid-like amniotic fluid. EBL was calculated based on the total amount of fluid, haematocrit value of the mixture, and the antenatal haematocrit of the patient.

Patients were divided into three groups according to EBL volume: general blood loss (GBL, EBL < 1000 ml), massive blood loss (MBL, 1000 ml ≤ EBL < 2000 ml), and extremely massive blood loss (ex-MBL, EBL ≥ 2000 ml). [16] The distribution of clinical factors in the three groups is outlined in Table 1.

### MRI analysis

#### MRI protocols

All subjects underwent MRI with a 1.5 T MR scanner (HDxt, GE Healthcare, City, State, USA), equipped with an eight-channel phased array coil for signal reception of the pelvic cavity. The patients were imaged in the supine or left side position according to their comfort. Fast imaging employing steady-state acquisition (FIESTA), single-shot fast spin-echo T2-weighted imaging (SSFSE T2WI), and liver acquisitions with volume acceleration (LAVA) were obtained in the axial, coronal, and sagittal planes. The acquisition parameters for all the sequences are listed in Table 2.

**Table 2:** MRI parameters

Parameter sequences	FIESTA	SSFSE T2WI	LAVA
TR/TE (ms)	3.6/1.6	1800/81	3.9/1.8
matrix size	224 × 256	288 × 192	288 × 200
Slice thickness (mm)	5–7	5–6	2.5
Slice gap (mm)	1	1	0.5
FOV (mm^2^)	400 × 400	380 × 380–400 × 400	400 × 400

*MRI* magnetic resonance imaging, *FIESTA* fast imaging employing steady state acquisition, *SSFSE: T2WI* single-shot fast spin-echo T2-weighted imaging, *LAVA* liver acquisition with volume acceleration, *TR* repetition time, *TE* echo time, *FOV* field of view

#### Description and analysis of two predictive MRI features

**Fig. 1:**
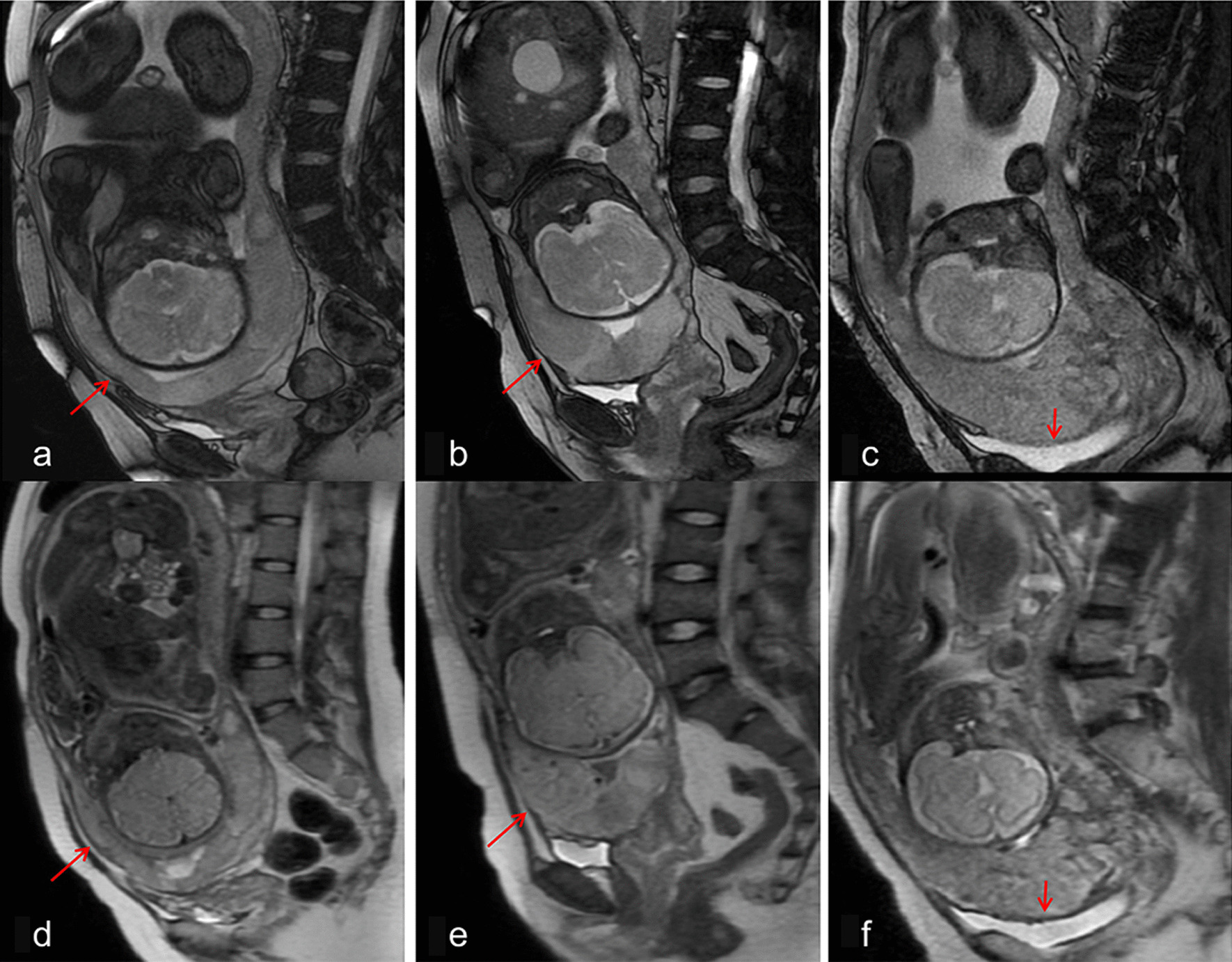
**a** FIESTA MRI showed continuous myometrium, with the signal of “low–high–low” trilaminar appearance, the thinnest part (arrow) measured at 2.6 mm. **b** FIESTA MRI showed that although the myometrium was continuous, the low-signal inner myometrial layer was unclear, the thinnest part (arrow) was less than 1.5 mm as measured by 2 radiologists. **c** FIESTA MRI showed the focal interruption (arrow) of myometrium, the low signal gap between the placenta and the bladder narrowed. **d**–**f** The SSFSE-T2WI sequences were illustrated for reference

The myometrium generally exhibits a typical trilaminar appearance in T2W images (FIESTA or SSFSE). It includes low-signal inner myometrial, high-signal outer myometrial, and low-signal serosal layers. When the myometrium thickness (MT) decreases, the trilaminar structure may be incomplete. Focal interruption of the myometrium can be described when the myometrium becomes discontinuous and is focally interrupted at the site of placenta bulging [17, 18]. FIESTA demonstrated a better outlineboundary and signal–noise ratio to illustrate clearly anatomical uterine structures [19]. For this reason, measurements and region-of-interest (ROI) drawings were completed in FIESTA images, and the SSFSE-T2WI was observed as a guide. Two radiologists attempted to measure the MT in consensus, twice at the thinnest site of the myometrium in the optimal axial, coronal, or sagittal plane of FIESTA before they estimated an average of the three values. However, there appears to be a special but not rare situation in our measurements. In some patients, thinning and continuous myometrium were observed in MR images; however, the thickness measured at the thinnest site was less than a numerical value. Thus, for this description, L_min_ was defined as the shortest edge length of the MRI voxel, which ranged from 1.5 to 2.0 mm. In these cases, the measurement errors were relatively large. Based on this situation, the radiologists were asked to grade MT. The classification was as follows: *grade 1 (G0)*, the focal interruption was observed in the myometrium; *grade 2 (G1)*, the myometrium was continuous, but the thinnest site was at locations < L_min_; and *grade 3 (G2)***,** the measured thinnest site of the myometrium was at locations ≥ L_min_. When the two radiologists reached different grading results, they negotiated and performed additional measurements more times to reach a consensus. The patients who fulfilled all three MT grade criteria are shown in Fig. 1.

DIB are defined as nodular or banded areas of low-signal intensity in the placenta on both the T2-SSFSE and the FIESTA sequences. DIB often appear on the mother side of the placenta [20]. For each patient, two radiologists selected the definable regions of DIB in consensus on the axial, coronal, and sagittal planes of FIESTA images. The edges of the DIB were then outlined by each radiologist. The picture archiving and communication system (PACS) automatically displays the areas of the DIB within the closed curves after the edges were outlined. The volume of the DIB was obtained by multiplying areas (the sum of the DIB area in the set of sectional images) and the MRI spacing (the sum of layer thickness and layer spacing) (Eq. 1). The first average DIB value was calculated from the results of three coronal, sagittal, and axial sections (Eq. 2).

1 $${\text{V}}p = ({\text{thickness}}p + {\text{gap}}p)\sum\limits_{i = 1}^{n} {Spi}$$

2 $${\text{V}}DIB = {\text{(Vaxial}} + {\text{Vsagittal}} + {\text{Vcoronal)/3}}$$

where V_p_ denotes the total volume of DIB, thickness_p_ is the slice thickness of MRI, and gap_p_ is the slice gap of the MR image.

**Fig. 2:**
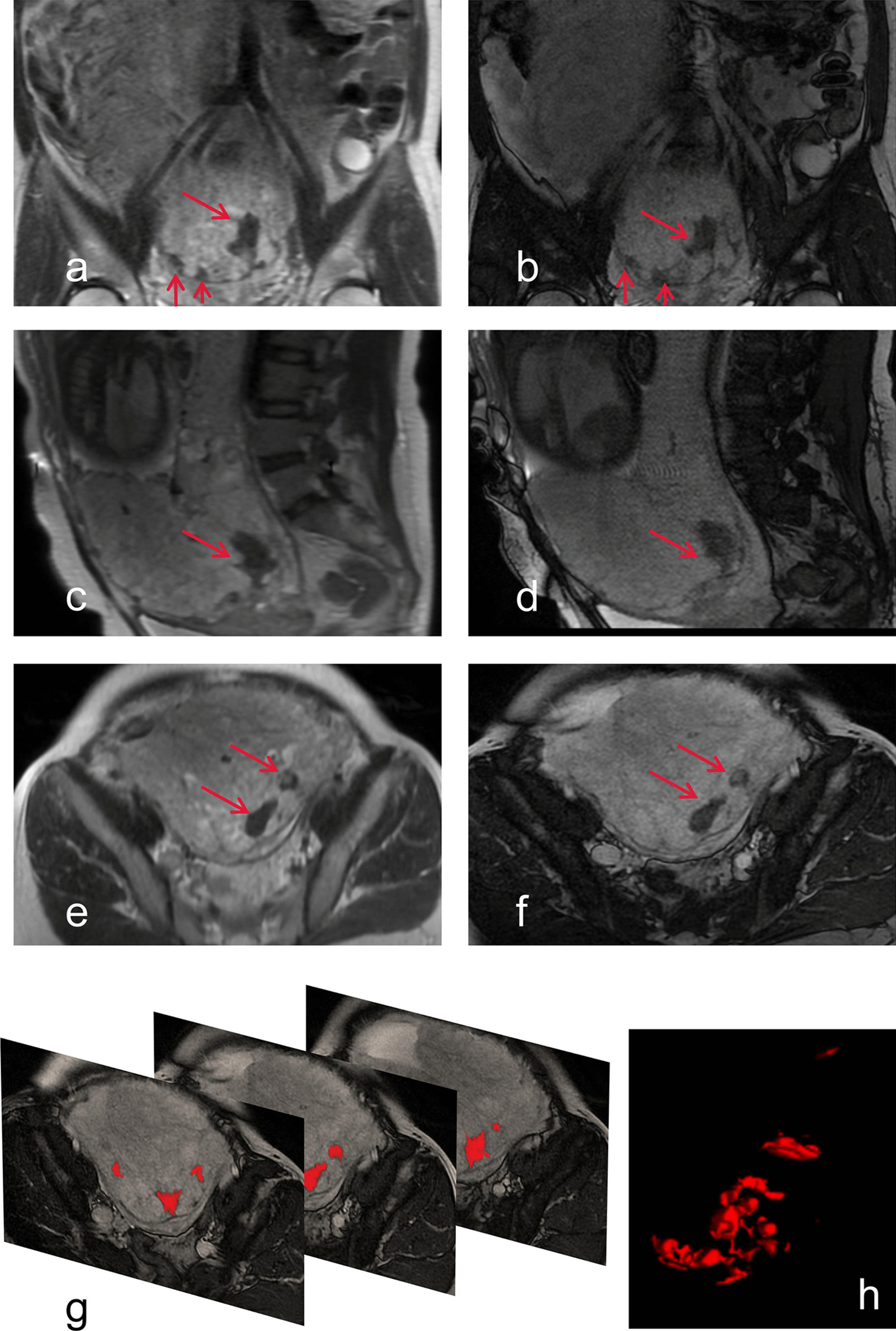
A woman with placenta accreta and had a estimated blood loss volume of 3000 ml. Preoperative MRI (33 weeks pregnant) showed the coronal (**a**, **b**), sagittal (**c**, **d**) and axis (**e**, **f**) planes, SSFSE (**a**, **c**, **e**,) and FIESTA (**b**, **d**, **f**) multiple dark intraplacental bands (arrow). **g** A demonstration of the dark intraplacental bands which was found then drawn on part of the axis plane; **f** A 3D reconstructed images was built by ITK-SNAP (Version 3.8.0) to demonstrate the dark intraplacental bands volume with the full images of the axis plane drawing

The final value was the average results of two radiologists. The DIB in the three sections and the demonstration of DIB volume measurement in part of axis images are shown in Fig. 2.

The location of placental attachment was judged by both radiologists. When the judgment was inconsistent, the two negotiated to determine the result. The placental attachment position was divided into five subtypes: (1) non-placental previa (non-PP), (2) low-lying placenta (LLP), (3) marginal placenta previa (MPP), (4) partial placenta previa (PPP), and (5) complete placenta previa (CPP) [21]. The radiologists were blind to the clinical and surgical data.

### Statistical analysis

Categorical data (history of CS or miscarriage, haemostasis, MT grade, placental attachment position subtype) were indicated according to frequency, and were analysed using the Chi-square test. The normality of variables for each data was detected by the Kolmogorov–Smirnov (K–S) test. Normally distributed data are indicated by *x* ± *s*, and were analysed by variance analysis. Non-normally distributed data were indicated by median values (first and third quartiles) and were analysed by using the multiple groups of independent sample rank sum test (Kruskal–Wallis).

The consistency of the two radiologists was validated by using kappa consistency analysis (for placental attachment positions and MT grades) and the interclass correlation coefficient (ICC) for DIB volumes.

The correlation analysis between the grade of MT, volumes of DIB, and EBL was performed with Spearman analysis; the Spearman correlation coefficient (ρ) was used to express the degree of correlation. The assessed efficacy of predicting severe PPH by the grades of MT and the volume of DIB was indicated by receiver operating characteristic (ROC) curves.

All statistical analyses and calculations were performed with SPSS (version 26, IBM, City, State, USA). *P* values < 0.05 were considered to be statistically significant.

## Results

### Clinical information

The general clinical information, preoperative blood information, history of CS and abortions, and haemostasis measurements are listed in Table 1. General clinical information, including age, gestational age, and time from MR scanning to CS showed no differences in the three groups. The RBC, coagulation routine examination, and the history of CS or abortion also showed no differences across the three groups. Among the six patients with no history of CS, three patients underwent 1 to 3 times of induced abortion operations, two patients underwent natural labour once, and one patient conceived through in-vitro fertilisation-embryo transfer (IVF-ET).

During the CS, different methods of haemostasis were used in each patient. There were some differences in the measures among the three groups (*P* = 0.014). Specifically, the proportion of patients who used prophylactic balloon occlusion was higher in the ex-MBL group than in the other two groups (*P* = 0.002).

### MRI features and the relation to EBL

**Fig. 3:**
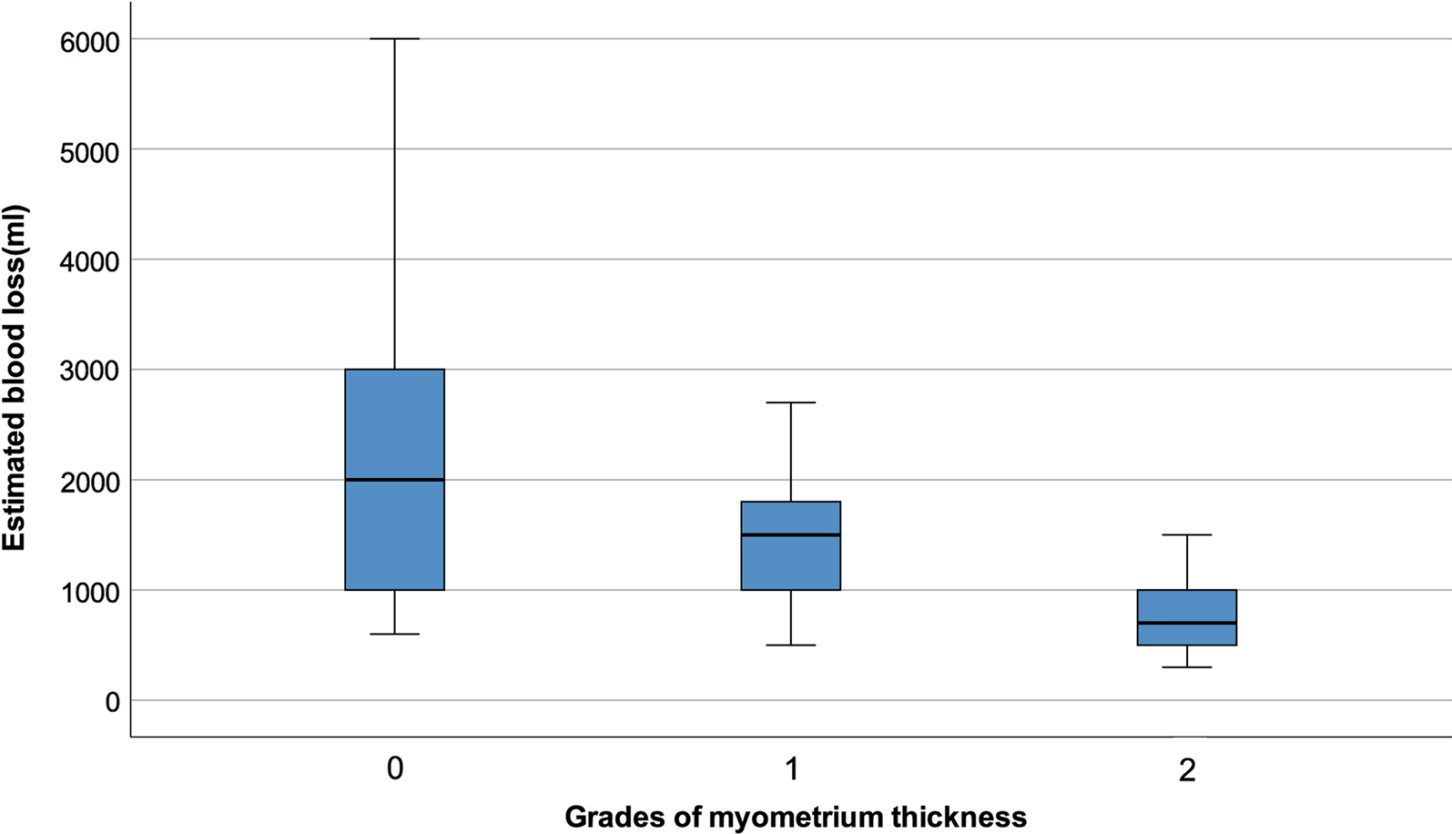
The distribution of estimated blood loss among the myometrium thickness grades (G0/G1/G2)

The kappa consistency analysis of MT grades by the two radiologists was 0.718. The MT measurements in all patients were: G0 = 37, G1 = 14, and G2 = 24. The distribution among the three EBL groups is shown in Table 3. The distribution of EBL between G0/G1/G2 is shown in Fig. 3. There was a significant negative correlation between MT grades and EBL (*P* < 0.001, ρ = − 0.604). (Table 3)

**Table 3:** The correlation between the myometrium thickness grades and dark intraplacental bands volume to the estimated blood loss and the amount of red blood cell input

	Estimated blood loss (ml)	Red blood cell input (u)	Myometrium thickness grades			Dark intraplacental bands volume (mm^3^)
			G0	G1	G2	
General blood loss (N = 25)	624.0 ± 156.2	0 (0,3.25)	5	1	8	242.85 (0, 4749.45)
Massive blood loss (N = 26)	1226.9.8 ± 172.1	4.0 (2.0,4.0)	11	10	14	4683.30 (0, 21,238.85)
Ex-Massive blood loss (N = 24)	2875.0 ± 1034.3	6.5 (4.0,9.5)	21	3	2	40,818.43 (19,281.72, 40,818.43)
*P* value	–	*P* < 0.001	*P* < 0.001			*P* = 0.007
Correlation to estimated blood loss (ml)	–	*P* < 0.001 ρ = 0.705	*P* < 0.001 ρ = − 0.604			*P* < 0.001, ρ = 0.653
Correlation to red blood cell input (u)	*P* < 0.001 rho = 0.705	–	*P* < 0.001 ρ = − 0.394			*P* < 0.001, ρ = 0.630

*u* unit

*Normally distributed data:* mean value ± standard deviation; *Non-normally distributed data:* median (first quartile, third quartile)

The ICC coefficient for DIB volume measurements by the two radiologists was 0.840. There was a significant positive correlation between DIB volume and EBL (*P* < 0.001, ρ = 0.653). (Table 3) In the MR images of 19 patients, DIB was not observed; therefore, the DIB volume was zero in these patients. The EBL of the 19 patients was 910.5 ± 448.3 ml. Regarding the remaining 56 patients, the DIB volume was 15,716.70 (4464.60, 36,435.83.90) mm^3^, and EBL was 1,771.4 ± 1,200.0 ml. These 56 individuals showed a significant positive correlation between DIB volume and EBL (*P* < 0.001, ρ = 0.697).

The kappa consistency analysis allowed the estimation of the placental attachment location by the two radiologists; this was equal to 0.778. The final results of the placental attachment location were: non-PP = 0, LLP = 6, MPP = 6, PPP = 3, and CPP = 60. Non-PP was removed from the subsequent analysis as no patients were in this group. Among the four placental attachment locations, there were no differences in the distribution of blood loss (*P* = 0.101) (Table 4).

**Table 4:** The distribution of blood loss among the placental attachment locations

	Placental attachment locations				Sum
	LLP	MPP	PPP	CPP	
*Blood loss*					
Group of GBL	1	3	2	19	25
Group of MBL	5	2	1	18	26
Group of ex-MBL	0	1	0	23	24
Sum	6	6	3	60	75

*GBL* general blood loss, *MBL* massive blood loss, *ex-MBL* extremely massive blood loss, *LLP* low lying placenta, *MPP* marginal placenta previa, *PPP* partial placenta previa, *CPP* complete placenta previa

**Fig. 4:**
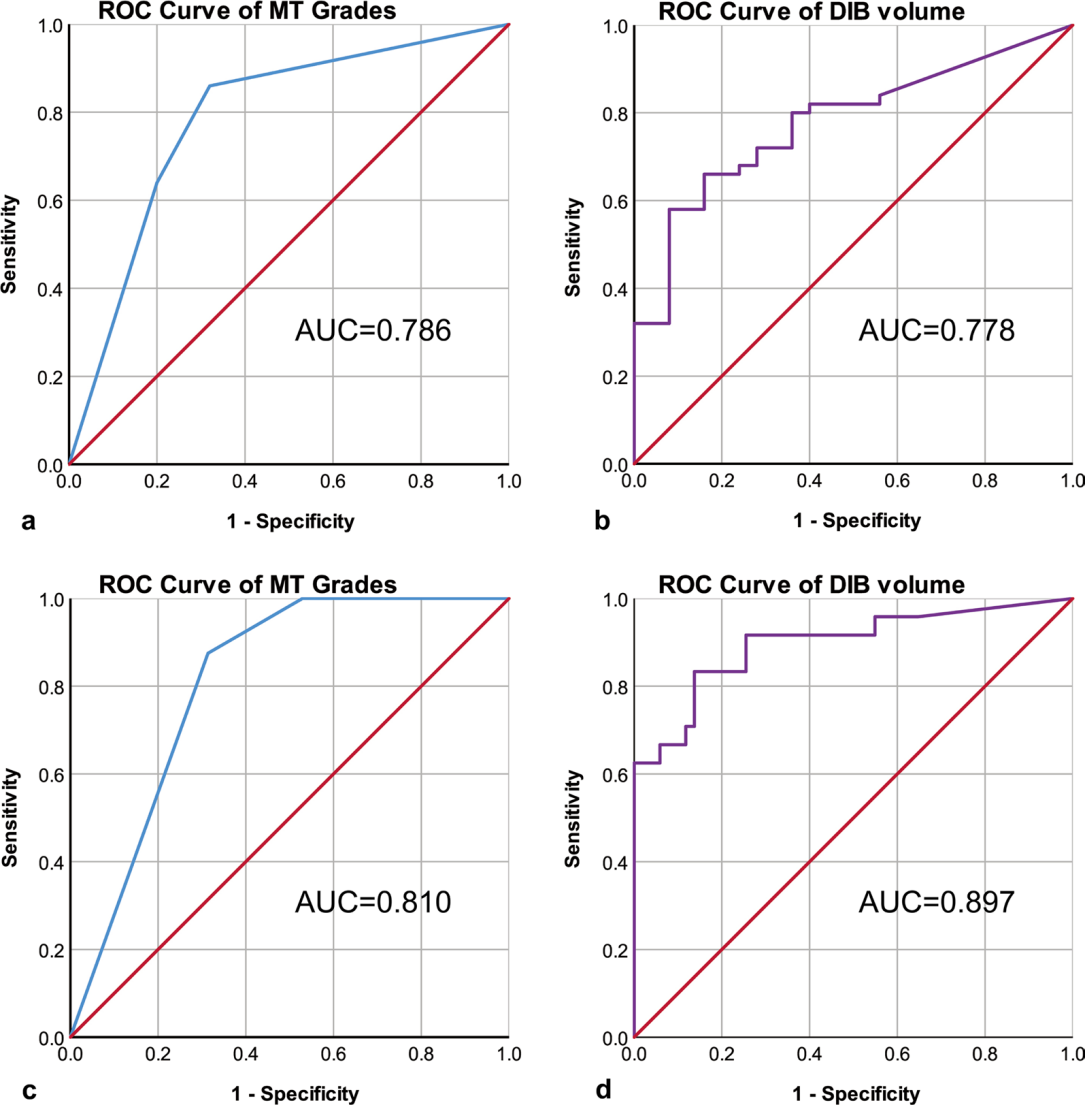
Receiver operating characteristic (ROC) curves for myometrium thickness grades (**a**), dark intraplacental bands volume (**b**) to predict the risk of massive postpartum haemorrhage (Estimated blood loss volume ≥ 1000 ml). ROC curves for myometrium thickness grades (**c**), dark intraplacental bands volume (**d**) to predict the risk of severe postpartum haemorrhage (estimated blood loss volume ≥ 2000 ml)

The ROC curves for MT grades and DIB volume for predicting massive haemorrhage (EBL ≥ 1000 ml) are shown in Fig. 4A, B. The areas under the curves (AUCs) were 0.786 and 0.778, respectively. The ROC curves for MT grades and volume of DIB for predicting severe haemorrhage (EBL ≥ 2000 ml) are shown in Fig. 4C, D. The AUCs were 0.810 and 0.897, respectively.

### Relationship between MRI features and depth of PAS invasion

There are three degrees of depth of PAS invasion: (1) placenta increta, (2) placenta accreta, and (3) placenta percreta. There was a significant difference in EBL among the three degrees (*P* < 0.001). A significant positive correlation was observed between EBL and the three degrees (*P* < 0.001, ρ = 0.493). There was no difference in MT among the three degrees of PAS invasion (*P* = 0.126), but regarding DIB volume, a significant difference could be found among the three degrees (*P* = 0.007). A significant positive correlation was observed between the volume of DIB and the three degrees (*P* = 0.002, ρ = 0.359) (Table 5).

**Table 5:** The correlation between the myometrium thickness grades and dark intraplacental bands volume to the depth of PAS invasion

	Estimated blood loss (ml)	Red blood cell input (u)	Myometrium thickness grades			Dark intraplacental bands volume (mm^3^)
			G0	G1	G2	
Placenta accreta (N = 15)	860.0 ± 671.7	0 (0,0)	6	3	17	242.85 (0, 4869.00)
Placenta increta (N = 44)	1481.8 ± 905.6	2.0 (0,4.0)	20	8	7	4464.60 (0, 14,476.35)
Placenta percreta (N = 16)	2400.0 ± 1479.6	6.9 (4.0,9.5)	11	3	0	26,192.48 (4788.38, 57,547.90)
*P* value	*P* < 0.001	*P* < 0.001	*P* = 0.126			*P* = 0.007
Correlation to the depth of PAS invasion	*P* < 0.001 ρ = 0.493	*P* < 0.001 ρ = 0.615	–			*P* = 0.002 ρ = 0.359

*PAS* placenta accreta spectrum

*Normally distributed data:* mean value ± standard deviation; *Non-normally distributed data:* median (first quartile, third quartile)

## Discussion

The selection of the two MRI features, MT and DIB, in this study was determined by the data and conclusions from the previous and recent literature. As this study on PPH was based on PAS, we studied several PAS-related MRI studies and focused on the results of systematic reviews and meta-analyses. Several studies aggregated multiple PAS-related MRI features, and used the presence or absence of these features as analytical indicators. In such studies, the two features selected yielded a high correlation with the depth of PAS invasion or PPH [9, 10, 22]. In another class of studies that established a predicting system for the maternal outcome based on PAS-related factors, including MRI features, the two selected features yielded high-score proportions [11, 23]. Furthermore, these two features have a higher frequency of occurrence in the MR images of PAS patients, which also constitutes the basis of the ability to perform grading and volumetric design.

The reproducibility of measurement might be time consuming. There could be some resolutions to address this flaw. For example, artificial intelligence could be used to realize a semi-automatic segmentation for the DIB curve. The segmentation of placenta in MR images was available [24], and we believe that this problem will be solved soon. Additionally, we believe that this research direction has good prospects.

MT (grades) showed a significant negative correlation with EBL. These findings indicated that as the thickness of the myometrium decreases, the risk of severe PPH increases. It is also worth mentioning that the difference in MT between the degree of depth of PAS invasion is not obvious. Therefore, it is not feasible to predict the depth of PAS invasion alone by MT. This can also be attributed to the thinning of the myometrium itself beyond the third trimester in PAS patients [25], with or without the CS scar. Hence, thinning was not significantly different between the three degrees of depth. The reasons why myometrium thinning (or interruption) correlated with PPH were as follows. First, myometrium thinning (or interruption) may be a direct sign of placental villus accreta into the myometrium in which increased blood loss was affected by intraoperative placental dissection difficulties caused by PAS. Second, regardless of the depth of invasion of the placental villus, it is difficult for thinner myometrium to produce an adequate uterine contractility during labour. When the contractions become weak, it is difficult to limit the maternal blood flow to the placenta bed during delivery, thus reducing the ability to control the bleeding in time [26].

Volumetry of DIB could be divided into two parts, namely, in the location of DIB, and outlining of the edges of DIB. The DIB location was based on consensus between the two radiologists. We used to design by completely double blinding the study as it pertained to the existence or absence of imaging features. There is no doubt that the double-blind method could increase the credibility of the results. However, in our study, the DIB volume was not assessed based on “existence or not”. When the two radiologists presented with significantly different results, they needed to re-examine the images to reach a consistent conclusion. In this process, we also considered that if we only re-discussed the cases with significantly different results, we might have missed some cases with small differences, but the DIB locations and delineation areas of the two radiologists were not completely consistent.

Hence, the locations of DIB were based on consensus between the two radiologists. Outlining of the edges of DIB was conducted independently by the two radiologists. The ICC coefficient was used to verify the consistency of the DIB volumes calculated by the two radiologists. The result of ICC coefficient showed the entirety were in good agreement.

Pathologically, DIB could be considered as fibrin foci deposited in placental tissue due to frequent bleeding and infarction [27, 28]. In this study, the volume of DIB exhibited a significant positive correlation with EBL and good sensitivity and specificity in predicting severe bleeding volume. We excluded the 19 patients without DIB features, to investigate whether the volume of DIB was associated with EBL when the MRI feature did exist; the results remained positive. This fully demonstrates that DIB volume can serve as a good indicator for predicting the degree of PPH. Moreover, a positive correlation between DIB volume and the invasion depth of PAS indicated that it also has the evaluation value for the degrees of the invasion depth of PAS. These results were similar to those reported in a previous study [29]. As a relationship exists between PAS invasion depth and EBL, we infer that this may be one of the factors by which DIB volume assesses the degree of bleeding. If further considered theoretically, excessive fibrin deposition foci will occupy the space in the placental villus space, while the uterine spiral arteries and draining veins will expand and increase to enrich the blood flow and blood volume of the placental bed. If the placenta is manually separated at delivery, the dilated and increased blood vessels may lead to the occurrence of a massive haemorrhage. Therefore, the volume of DIB exhibited high feasibility as a quantitative predictive indicator of the degree of PPH.

Clinical and US criteria can screen out the target patients who were suspected to be affected by PAS. However, preoperative clinical and US evidence could not be used as strong evidence for PAS diagnosis. Therefore, the intraoperative PAS diagnosis was also used as an inclusion criterion.

Each patient in the study underwent elective caesarean section. The surgical protocol was developed in advance for each patient. The EBL may be associated with some errors during the collection of liquid or during calculation. Hence, we also analysed the correlation of our imaging features and the transfusion of RBC. We believe the transfusion could also reflect the patients’ status. Given the RBC input can also indirectly reflect PPH, the correlation between the MT grading and volumetry of the DIB, was also investigated. Both MT grades and the volume of DIB correlated with the RBC input, but the volume of DIB exhibited a more significant relationship. This has high-clinical significance and means that the reasonable range of intraoperative blood product reserves can be evaluated by using these two features before surgery.

The history of CS and abortion exhibited no differences among the three blood loss groups. There were also no significant differences between the preoperative blood routine and coagulation indices among the three groups. This means that none of the above clinical factors had a significant impact on the differences in blood loss.

In addition, patients underwent more prophylactic balloon occlusions in the group ex-MBL, which was somewhat different from the other two groups; however, the procedure was aimed at intraoperative haemostasis, and not to the occurrence of massive blood losses. Therefore, it is not an indicator that affects the conclusion of this study.

Further, there were no significant differences among the three EBL groups regarding the subtypes of placental attachment positions in this study, probably owing to the excessive proportion of patients with CPP (75.80%); therefore, it is difficult to summarise the trends that affect blood loss.

This study is associated with some limitations and deficiencies. First, we are pleased to conclude that MRI features could predict the PPH in PAS patients; however, we believe people would prefer to see that MR is able to assess the risk of PPH based on a large amount of normal puerpera. Therefore, given that MRI has not been extensively used yet, more data may be collected if the convenience and safety of MRI are improved. Second, some other MRI features, such as placental/uterine bulge, bladder wall interruption, abnormal placental, subplacental vessels, and altered placental heterogeneity [30, 31], were not analysed in this study. However, one or more of them had been considered to provide valid evidence for diagnostic PAS in the previous literature. We will look for appropriate quantitative ways to explore the relationship between other MRI features and blood loss in the future. Third, the sample size included was relatively small; therefore, more patients need to be enrolled to enrich the study results in the future.

## Conclusion

The grading and volumetric preoperative MRI features, MT grades, and DIB volume can be used as good indicators to predict the risk of massive bleeding in CS. The numerical values of the two indicators based on the preoperative MR images can guide the decision of blood product reserve and surgical mode.

## Data Availability

The datasets generated and/or analysed during the current study are not publicly available due the further research is needed based on current results, but are available from the corresponding author on reasonable request.

## Abbreviations

CS: Caesarean section
DIB: Dark intraplacental bands
EBL: Estimated blood loss
FIESTA: Fast imaging employing steady-state acquisition
IVF-ET: In-vitro fertilisation-embryo transfer
MRI: Magnetic resonance imaging
MPP: Marginal placenta previa
MT: Myometrium thickness
non-PP: Non-placental previa
PAS: Placenta accreta spectrum
PPH: Postpartum haemorrhage
ROC: Receiver operating characteristic
US: Ultrasound
